# Supplementary material for: Challenges in predicting PROTAC-mediated protein–protein interfaces with AlphaFold reveal a general limitation on small interfaces
Source: Bioinform Adv. 2025 Mar 14;5(1):vbaf056. doi: 10.1093/bioadv/vbaf056 (PMC11938821; doi:10.1093/bioadv/vbaf056)
Supplement: vbaf056_Supplementary_Data [file vbaf056_supplementary_data.docx]

Supplementary Information

Table S1. PDB Ids used in this study. Numbers in parentheses refer to the number of cases.

| Dataset | Ids |
| --- | --- |
| PROTACS (28) | 5HXB 5T35 6BN7 6BOY 6HAX 6HAY 6HR2 6W7O 6W8I 6XK9 6ZHC 7JTO 7JTP 7KHH 7LPS 7PI4 7Q2J 7S4E 7Z6L 7ZNT 8BB2 8BB3 8BB4 8BB5 8BDS 8BDT 8BDX 8BEB |
| Test set, no ligand (321) | 7G80 7PYV 7Q1U 7Q4H 7Q72 7QBM 7QE5 7QE8 7QGS 7QIH 7QLA 7QUU 7R2E 7R49 7SH3 7SHQ 7SJ4 7SJQ 7SL9 7SO0 7SR4 7SVT 7SZA 7T0Y 7T5P 7T7I 7TA3 7TCR 7TJ4 7TL7 7TPB 7TVJ 7TYD 7TZH 7U20 7U5O 7U5P 7UBZ 7UCC 7UFW 7UJ3 7UL2 7ULA 7UM4 7UPM 7URV 7VLJ 7VRC 7VUO 7VVB 7VW6 7W3U 7W47 7WA4 7WDK 7WFC 7WGE 7WKI 7WKO 7WMV 7WN0 7WQU 7WQZ 7WRS 7X3K 7X9S 7XAD 7XAE 7XDD 7XEY 7XGE 7XKY 7XLD 7XPC 7XQK 7XQW 7XVG 7XVK 7XYQ 7Y1Q 7Y4A 7Y6B 7Y8U 7Y9N 7YAG 7YCJ 7YCO 7YGG 7YK3 7YKA 7YLD 7YLS 7YN3 7YO3 7YR5 7YTT 7YUI 7YZ9 7Z3Q 7Z71 7Z72 7Z7E 7ZAU 7ZCG 7ZCL 7ZCM 7ZD5 7ZEY 7ZH3 7ZHR 7ZJM 7ZJU 7ZSC 7ZYU 8A1G 8A4A 8A82 8A8C 8A8F 8A8M 8ADB 8AIM 8AIN 8AIW 8AJ8 8AJN 8AJY 8AKO 8ALK 8ALO 8ALS 8ALZ 8APY 8AV2 8AW4 8B05 8B08 8B0U 8B2N 8B2Q 8B3S 8B5S 8B8V 8BA1 8BAN 8BB7 8BCT 8BE2 8BFJ 8BGM 8BJH 8BMX 8BOS 8BOZ 8BUZ 8BV0 8BVR 8BW8 8BWF 8BYP 8BZR 8C0J 8C26 8C3K 8C44 8C47 8C5H 8CD0 8CJB 8CQI 8CQZ 8CT8 8CUS 8CUU 8CWS 8CXJ 8CXR 8D4S 8D5V 8DC0 8DD7 8DLE 8DML 8DMQ 8DRN 8DSO 8DY1 8DYE 8DYO 8DYP 8E0E 8E1D 8EFP 8EFW 8EFX 8EHE 8EHO 8ENB 8EXE 8EY4 8EZL 8EZR 8F0M 8F19 8F6D 8F7A 8FFU 8FHK 8FJ2 8FN8 8FN9 8FZZ 8G0P 8G4Y 8GBQ 8GJA 8GKO 8GP6 8GQE 8GRJ 8GS1 8GTG 8GUO 8GXE 8GZV 8H1I 8H3U 8H3X 8H62 8H6S 8H8A 8HBN 8HBV 8HCI 8HF3 8HFC 8HFP 8HGJ 8HHJ 8HKH 8HL7 8HLM 8HM3 8HSB 8HTC 8I2E 8I3G 8I4E 8I6J 8I8Y 8I9M 8I9Q 8IF0 8IJ9 8IN9 8IPQ 8IPS 8ITP 8IZN 8J09 8J1R 8J64 8JCA 8JMQ 8JO3 8JTK 8JTW 8JWJ 8K4R 8ODO 8OI2 8OI3 8ONT 8OOF 8ORN 8OYP 8P98 8PFC 8PIJ 8PKC 8PNL 8PQ7 8Q00 8Q5R 8Q66 8QF4 8QVW 8R4C 8R4O 8R7A 8R8Q 8RCQ 8RGI 8SBB 8SJJ 8SM0 8SNL 8SO9 8SSM 8ST7 8ST8 8ST9 8SYM 8T12 8T1C 8TJ3 8TWD 8U45 8UH1 8VOH 8VOI 8VX9 8WNS 8WQR 8WTB 8WWY 8X6B 8X77 8X8T 8XLD 8XY7 |
| Test set with ligand (5) | 7WR6 7XJP 7ZHM 8FDB 8OW6 |
| Training set with ligand (20) | 1ZHH 2EJF 2FYD 3EJD 3N7R 3NY7 3P71 3ZDF 4A69 4ETW 4F48 4I6N 4IG7 4JQU 4MRT 4PWV 5CZD 5EJD 5GPG 5KP8 |

Table S2. Repartition of cases by interface size range (expressed in Å^2^) and model quality (assessed by the DockQ score). Numbers in parentheses correspond to numbers of cases.

|  | Mean DockQ score | DockQ score < 0.23 | DockQ score >=0.23 |
| --- | --- | --- | --- |
| ΔASA<500 | 0.07 | 92% (12) | 8% (1) |
| 500≤ΔASA<1000 | 0.16 | 76% (25) | 24% (8) |
| 1000≤ΔASA<1500 | 0.46 | 43% (31) | 57% (41) |
| 1500≤ΔASA<2000 | 0.46 | 42% (26) | 58% (36) |
| 2000≤ΔASA<2500 | 0.54 | 25% (17) | 75% (50) |
| 2500≤ΔASA<3000 | 0.59 | 18% (7) | 82% (32) |
| 3000≤ΔASA<3500 | 0.64 | 11% (2) | 89% (16) |
| 3500≤ΔASA<4000 | 0.66 | 6% (1) | 94% (17) |
| 4000≤ΔASA<6000 | 0.57 | 22% (8) | 78% (28) |
| 6000≤ΔASA | 0.55 | 6% (1) | 94% (15) |


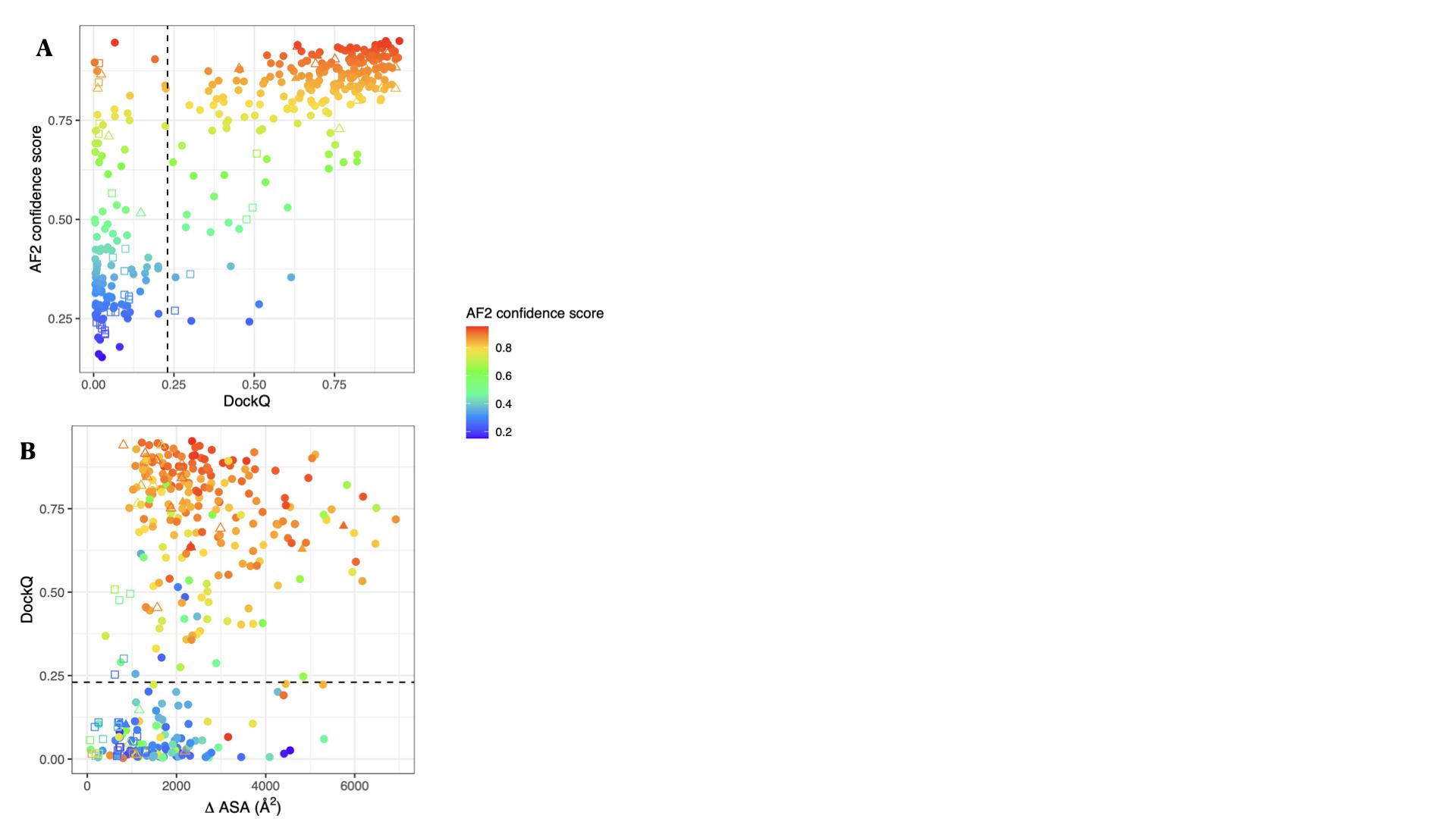


Figure S1. Model quality, AF2 confidence score and interface size. Points are colored according to the AF2 confidence score (0.8 ipTM+0.2pTM).


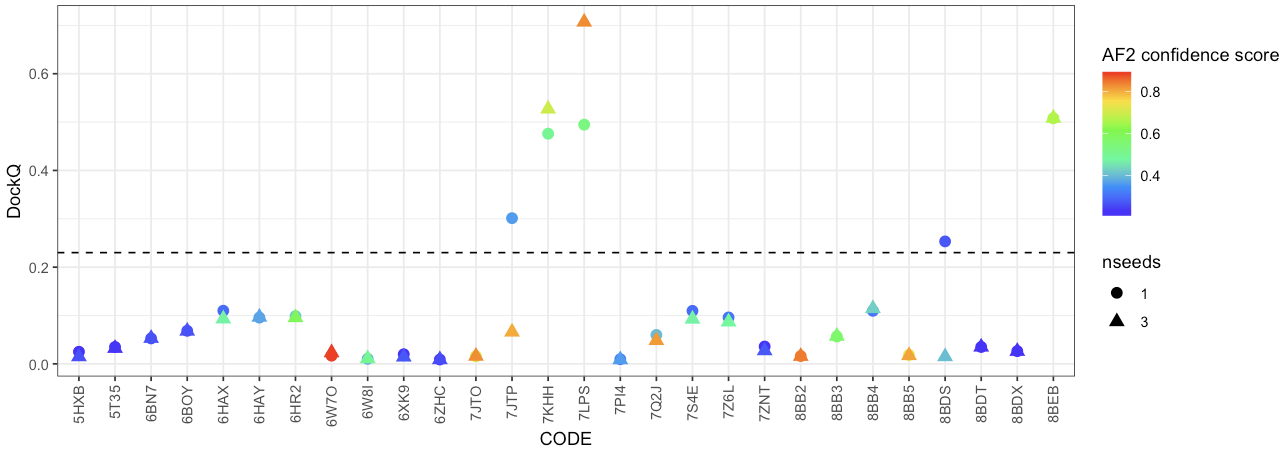


Figure S2. Model quality and AF2 confidence score in the PROTAC set, with 1 and 3 seeds. Points are colored according to the AF2 confidence score (0.8 ipTM+0.2pTM), and the shape indicates the number of seeds used for AF2 calculation.


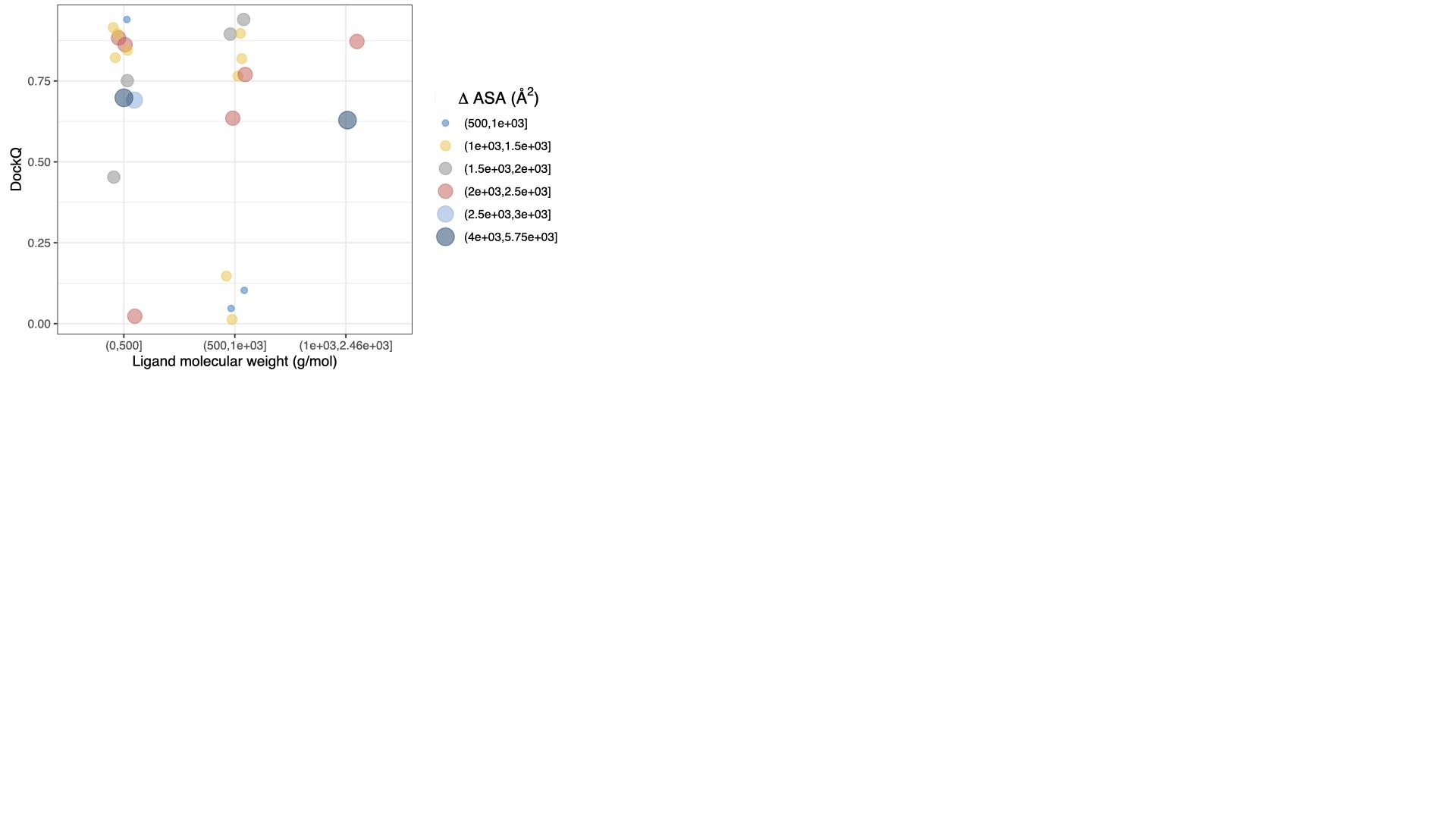


Figure S3. DockQ score as a function of ligand molecular weight for the 25 ligand-mediated complexes (not PROTACs-mediated complexes). Four out of five wrong predictions are for interfaces mediated by ligands with high molecular weight (>500g/mol). Note that these wrong predictions also involve small interfaces (small circles), and that the majority of complexes involving ligands of high molecular weight are well predicted.

*
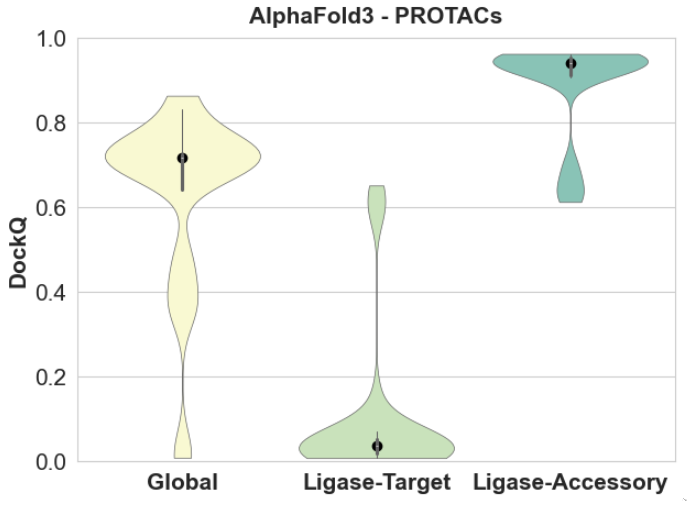
***Figure S4** - AlphaFold3 results for the 28 PROTAC-mediated complexes. In this experiment, all proteins within the PDB entry were provided to AlphaFold server and all 5 top-predicted models were queried against their corresponding crystal structure. We extracted the best ranked solution to AF3.

The data in Figure S4 show that AF3 is apparently performing at an acceptable level for the full systems, with an average DockQ score of 0.75. However, once these predictions are separated into two categories, the Ligase-Target interface DockQ and the PPIs involving the ligase and accessory proteins, we see the same pattern as in AF2-Multimer. In general, Ligase-Target interfaces remain difficult to predict with AF3, with only 4 systems out of 28 achieving a DockQ score above 0.23 for at least one predicted structure. This result is only marginally better than the result obtained with AF2, where three out of 28 systems were correctly predicted. In contrast, all interfaces not including the target protein were well predicted (all above Dock > 0.6) and the average DockQ score was around 0.9. This data paint a concerning picture: While on the surface these multi-protein complexes appear to be correctly predicted by AF3, it turns out that the most important interface is still missed in the large majority of the cases.


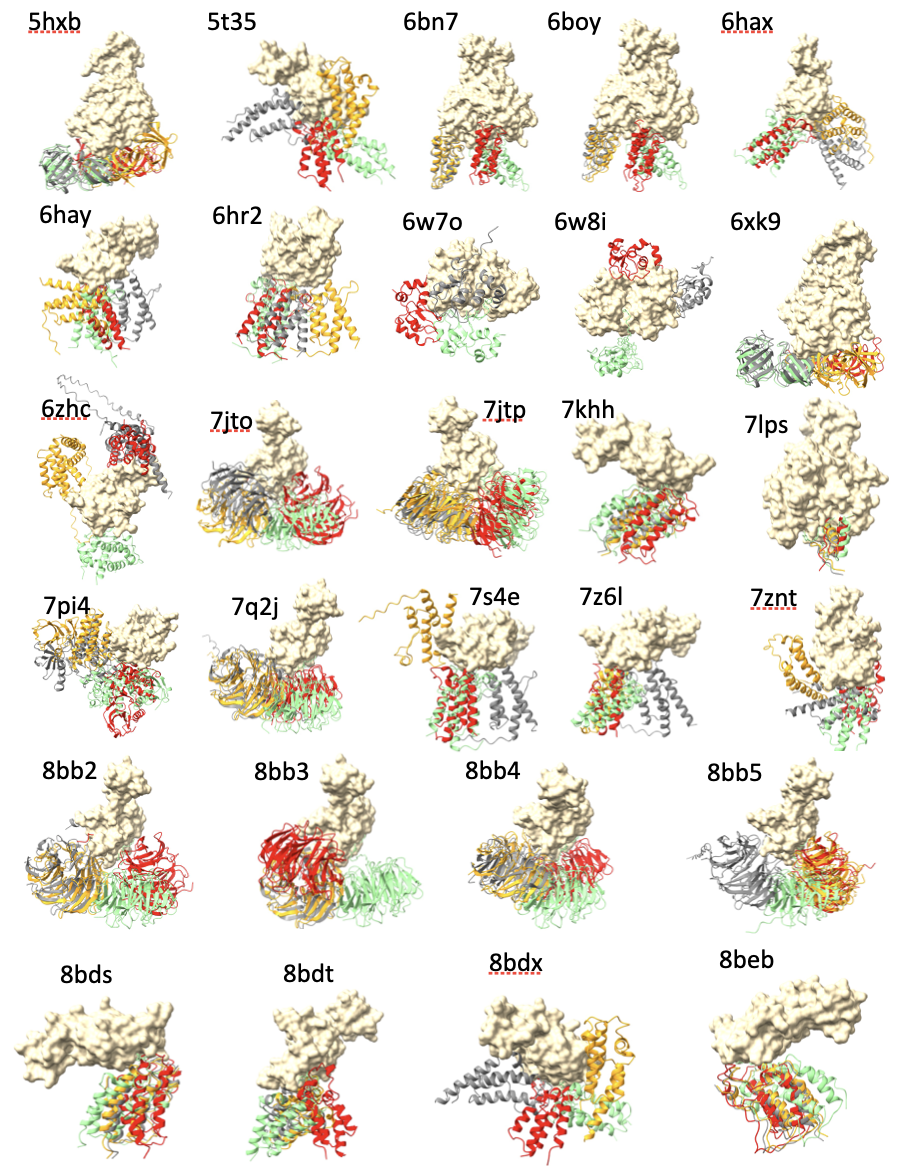


**Figure S5 - AlphaFold Models for the 28 PROTAC cases.** The E3 ligases are shown in surface representation and the protein targets in cartoon representation with the following color code: green=experimental structure, red=AF2 prediction, gray=AF3 prediction, orange=AF3 prediction with context.
